# Coronavirus conspiracy beliefs, mistrust, and compliance: taking measurement seriously

**DOI:** 10.1017/S0033291720005164

**Published:** 2020-12-10

**Authors:** John Garry, Rob Ford, Rob Johns

**Affiliations:** 1 Queen's University Belfast, Belfast, Northern Ireland; 2 University of Manchester, Manchester, UK; 3 University of Essex, Colchester, UK

**Keywords:** Coronavirus conspiracies, public opinion, mistrust, compliance, survey design

## Abstract

**Background:**

Freeman *et al*. (2020a, *Psychological Medicine*, 21, 1–13) argue that there is widespread support for coronavirus conspiracy theories in England. We hypothesise that their estimates of prevalence are inflated due to a flawed research design. When asking respondents to their survey to agree or disagree with pro-conspiracy statements, they used a biased set of response options: four agree options and only one disagree option (and no ‘don't know’ option). We also hypothesise that due to these flawed measures, the Freeman et al. approach *under*-estimates the strength of the correlation between conspiracy beliefs and compliance. Finally, we hypothesise that, due to reliance on bivariate correlations, Freeman et al. *over*-estimate the causal connection between conspiracy beliefs and compliance.

**Methods:**

In a pre-registered study, we conduct an experiment embedded in a survey of a representative sample of 2057 adults in England (fieldwork: 16−19 July 2020).

**Results:**

Measured using our advocated ‘best practice’ approach (balanced response options, with a don't know option), prevalence of support for coronavirus conspiracies is only around five-eighths (62.3%) of that indicated by the Freeman et al. approach. We report mixed results on our correlation and causation hypotheses.

**Conclusions:**

To avoid over-estimating prevalence of support for coronavirus conspiracies, we advocate using a balanced rather than imbalanced set of response options, and including a don't know option.

## Introduction

*Psychological Medicine* recently published a paper titled *Coronavirus conspiracy beliefs, mistrust, and compliance with government guidelines in England* (Freeman et al., 2020a). The paper argues that there is widespread support for coronavirus conspiracy theories in the general population of England, and that this may hinder public compliance with UK government advice/instruction on appropriate behaviour during the ongoing coronavirus 2019 (Covid-19) pandemic. The findings have been widely publicised by the authors and their university in a press release (University of Oxford, 2020), leading to headlines such as ‘One fifth of English people in study blame Jews or Muslims for Covid 19’ (Newsweek, 2020).

We strongly agree with Freeman et al. (2020a) about the importance of this topic. However, consistent with concerns expressed by McManus, D'Ardenne, and Wessely (2020) and Sutton and Douglas (2020), we hypothesise that the core claim in their paper about the prevalence of belief in coronavirus conspiracy theories is flawed because Freeman et al. (2020a) use a highly problematic measure of public support for conspiracy beliefs. In their survey, respondents were asked to either agree or disagree with a range of coronavirus conspiracy statements by indicating one or other of the following options: ‘do not agree’, ‘agree a little’, ‘agree moderately’, ‘agree a lot’ or ‘agree completely’. This is an imbalanced set of response categories, with four of the five categories representing support for the conspiracy theories and only one representing opposition. It makes agreement seem the norm and disagreement the exception. There is thus a significant risk, using this set of response options, of over-estimating the prevalence of support for Covid 19 conspiracies in the general population, and hence providing a false account of public attitudes.

A core tenet of questionnaire design is to aim for neutrality. The fact that response effects can be triggered by the subtlest of wording differences (e.g. Schuman & Presser, 1996) only underlines the need to avoid more blatantly leading designs. Unbalanced response scales like the one used by Freeman et al. (2020a) are therefore extremely rare. There is one exception worth noting: the measurement of happiness. Because the distribution of self-reported happiness is skewed such that most respondents are in the positive range of the scale, some researchers have experimented with scales that offer more options on that side of the neutral point (Kalmijn, Arends, & Veenhoven, 2011; Liao, 2014). If it were well established that clear majorities endorsed Covid 19 conspiracies, there may be a case for offering more options to differentiate shades of that agreement. But that is not established – indeed, polls point to conspiratorial reasoning being a minority position (even if sometimes a troublingly large minority). Even if it were, those using unbalanced scales recommend offering at least two points on each side of the neutral point (Liao, 2014).

Another reason for imbalance might be to encourage respondents to endorse what they might reasonably see as socially undesirable or counter-normative beliefs (Paulhus, 2018). It is hard to test *a priori* the assumption underlying this choice, namely that the greater danger is respondents with genuinely conspiratorial beliefs concealing them rather than respondents without strong beliefs being led by a biased response scale. However, decades of research confirming the shallowness and malleability of public opinion (Converse, 1964; Zaller & Feldman, 1992) point to the latter. This is presumably why survey methodologists do not include unbalanced response scales when listing standard techniques for trying to mitigate social desirability bias (Nederhof, 1985).

An additional problem with the Freeman et al. (2020a) response categories is the lack of an option for respondents to indicate that they have no opinion or don't know. Unlike imbalanced response scales, non-response options are something of a grey area in survey methodology and omitting ‘don't know’ can be reasonable when measuring pure opinions, which require little knowledge *per se*. However, it is not generally recommended for measuring beliefs about the state of the world, especially in the scientific realm (Tourangeau, Maitland, & Yan, 2016). It also exacerbates the problem of imbalance. If respondents who are genuinely unsure are forced to answer off the top of their head (see Baka, Figgou, and Triga, 2012), they are more likely to choose a middle option – typically a neutral category but, in the case of the Freeman et al. (2020a) set of response options, an ‘agree’ category.

Finally, the set of statements that Freeman et al. (2020a) ask their respondents to agree or disagree with are all statements in support of a conspiracy theory. This exacerbates the problem of acquiescence bias whereby respondents may tend, in general, to agree with statements rather than disagree with them (McClendon, 1991; Pasek & Krosnick, 2010)^†^^1^, again potentially boosting declared support for the pro-conspiracy statements. A more substantively balanced overall measure of attitudes to coronavirus beliefs would include approximately equal numbers of statements supportive of conspiracies and statements either supporting official explanations or dismissing conspiratorial explanations of coronavirus. This has two benefits. First, it makes the overall scale a purer measure of the targeted attitudes because acquiescence bias largely cancels out across the equal number of pro-conspiracy and anti-conspiracy statements. Second, whichever items are eventually selected for scaling, a balanced set of items eases respondent suspicions that the researchers have an agenda – either for or against conspiracies – and thus makes any given item a more neutral measure of attitudes (Billiet, 1995).^2^

Our concerns have been echoed by the Statistical Ambassador of the Royal Statistical Society, Anthony B. Masters, who has argued that the Freeman et al. (2020a) analysis is likely to overestimate the prevalence of support for coronavirus conspiracies because of an imbalanced scale, lack of neutral option and acquiescence bias:

This is an unusual set of options. There are four options to agree, and only one to disagree. There is no means to express having a neutral or no opinion … The possible responses should balance between agreement and disagreement … There is acquiescence bias. This is where people agree to a statement, no matter what it says. Some people are agreeable, or want to complete the survey. Such questions are easy to write, but may not give a true reflection of public attitudes … Imbalanced options and acquiescence bias means likely overestimation of agreement. This survey may overestimate how many English adults hold conspiratorial beliefs. Question wording and response options matter. Researchers should give balanced response options. Press reports about single questions should refer to the survey's imbalanced options. (Masters, 2020)

We offer an improved measure of the prevalence of support for coronavirus conspiracies that we believe provides a more accurate portrayal of public beliefs. First, following best practice, instead of the imbalanced set of response options offered by Freeman et al. (2020a), our measure includes a symmetrical and balanced set of response options: strongly disagree, disagree, slightly disagree, slightly agree, agree, strongly agree. Second, following best practice, our measure offers respondents a ‘don't know’ option for each statement. Third, following best practice, our measure presents respondents with an approximately equally balanced set of statements with which to agree or disagree: some of the statements are supportive of coronavirus conspiracies and some are consistent with the ‘true’ or official interpretation of coronavirus.

In our pre-registered study, we conduct a survey of the English population and ask a representative sample of approximately 1000 respondents our ‘best practice’ survey questions. Our contention is that this will elicit a more accurate description of public beliefs than that reported by Freeman et al. (2020a). In order to demonstrate exactly *why* the prevalence levels reported by Freeman et al. (2020a) are likely to be over-estimates, we include an experimental component in our survey. Our experiment focuses on the main flaw in the Freeman et al. (2020a) approach – specifically, the response options they use. Alongside the ‘best practice’ format described above, we add two further conditions. The first condition replicates the Freeman et al. (2020a) approach. Approximately 500 people receive the same set of questions but with the exact response options (skewed in a positive direction) used in the original Freeman et al. (2020a) study. The second condition reverses the imbalance: approximately 500 people receive the same questions but with response options skewed in a *negative* direction. The total number of respondents to our survey (~2000) are thus randomly allocated in a 2:1:1 proportion among the three conditions: ‘best practice’, ‘positive skew’ and ‘negative skew’.^3^

We hypothesise that agreement with coronavirus conspiracy statements will be significantly lower in the ‘negative skew’ than the ‘positive skew’ condition. If confirmed, this would provide direct experimental evidence showing that ‘skewing’ response options when measuring support for coronavirus conspiracy statements has an effect on the results.

**Hypothesis 1**: Agreement with Coronavirus conspiracy statements is higher when response options are positively skewed (four ‘agree’ categories and one ‘disagree’ category) than when response options are negatively skewed (four ‘disagree’ categories and one ‘agree’ category’)

We further hypothesise that agreement levels for the coronavirus statements in our ‘best practice’ condition will be lower than in the ‘positive skew’ condition. This would demonstrate that having balanced substantive response options and a ‘don't know’ option leads to lower prevalence estimates than using a positive skew response set, as used by Freeman et al. (2020a).

**Hypothesis 2**: Agreement with Coronavirus conspiracy statements is higher when response options are positively skewed (four ‘agree’ categories and one ‘disagree’ category) than when response options are balanced (three ‘agree’ and three ‘disagree categories) and also include a ‘don't know’ option.

Also, we expect that if we exclude the ‘don't’ know’ respondents from our ‘best practice’ condition in order to simply focus on those offering a substantive response as is the case in the other two conditions, our prevalence estimates will fall between the ‘negative skew’ and ‘positive skew’ conditions.

**Hypothesis 3**: Agreement with Coronavirus conspiracy statements using a balanced set of response options three ‘agree’ and ‘three disagree’ is (a) lower than when using response options that are positively skewed (four ‘agree’ categories and one ‘disagree’ category), and (b) higher than when using response options that are negatively skewed (four ‘disagree’ options and one ‘agree’ option).

In addition to seeking to offer a more accurate portrayal of the *prevalence* of coronavirus conspiracy beliefs, our study also focuses on the *relationship* between conspiracy beliefs and compliance with government guidelines and recommendations on public behaviour to limit the spread of the virus. We expect to observe, as Freeman et al. (2020a) did, a significant bivariate relationship between pro-conspiracy beliefs and reported compliance. Given that we use a ‘best practice’ approach to measuring conspiracy beliefs we expect that we will observe a *stronger* bivariate relationship between conspiracy beliefs and compliance than that observed by the Freeman et al. (2020a) approach. This is because Freeman *et al*.'s (2020a) measures conflated all intensities of disagreement with conspiracy statements into a single category and hence were blunt instruments for measuring variation, and also forced ‘don't know’ respondents into a substantive category when they may not really have the attitude associated with that category.

**Hypothesis 4**: The bivariate relationship between support for Coronavirus conspiracy statements and compliance with Coronavirus guidelines is stronger when the multi-item conspiracy scale is generated from items with balanced response options and a don't know option than when the multi-item conspiracy scale is generated using imbalanced response options and no ‘don't know’ option.

Our final hypothesis is about one of the preconditions for making any causal claims about the relationship between coronavirus conspiracy beliefs and compliant behaviour. Causal inference requires (among other things) a model of compliance that also includes other obvious and simple explanatory factors that are likely to be related to conspiracy beliefs and offer a plausible explanation of compliance. Specifically, and in line with Freeman et al. (2020a), we focus on the concept of mistrust. However, while they only examined bivariate relationships, we hypothesise that if a general measure of trust in political institutions and medical professionals is introduced *into the same model* as a measure of support for conspiracies, the apparent effect of the latter will reduce significantly. Our suspicion is that non-compliance will often be a function of low trust in those requesting compliance rather than a function of support for outlandish and complex conspiratorial interpretations of the virus. This suspicion is worth testing given the important policy implications of the findings reported by Freeman et al. (2020a). They suggest that conspiracy beliefs may be causally related to non-compliance but, insofar as the underlying driver of non-compliance is simple mistrust, then responses focused on combating conspiracy theories will be directed at the wrong target.

**Hypothesis 5**: The strength of the relationship between support for Coronavirus conspiracy beliefs and compliance with Coronavirus guidelines significantly reduces once a general measure of trust is introduced into the model.

## Methods

### Data

We commissioned Deltapoll, an established British online survey agency, to conduct a survey of a representative sample of 2000 of the England population (achieved *n* = 2057; fieldwork 16−19 July 2020). Deltapoll invited members of online panels to participate. Quotas were applied to the following variables: gender, age, Government Office Region and 2019 vote. Data are weighted to a number of geo-demographic and voter-graphic variables. First, data are weighted to match demographic population targets from reliable sources (such as population estimates from the Office for National Statistics, the National Readership Survey, or the Labour Force Survey): data are weighted by age within gender, social class, household tenure work status, Government Office Region, and educational attainment. Further sets of weights are applied for recall of 2019 General Election vote, recall of 2016 EU referendum vote, and political attention (10-point scale weighted to British Election Study face-to-face survey). Our sampling and weighting approach thus yields a representative sample of the England population and is similar to the approach adopted by Freeman et al. (2020a). After the sample was initially recruited to participate, just over 1% (28 out of 2085 respondents) declined to continue once it was explained that the survey included some detailed questions about different interpretations of coronavirus (a similarly low discontinuation rate to that reported by Freeman et al., 2020b).

### Measures

#### Measuring prevalence of support for conspiracies: experimental design

Respondents are randomly allocated to one of three conditions. All three conditions contain 10 identical statements with which respondents are asked to either agree or disagree. The response options vary across the experimental conditions. In condition 1, the response options are positively skewed (as per Freeman et al., 2020a): do not agree, agree a little, agree moderately, agree a lot, agree completely. In condition 2, the response options are negatively skewed and are the opposite of the Freeman et al. (2020a) options: disagree completely, disagree a lot, disagree moderately, disagree a little, agree. In condition 3, the ‘best practice’ condition, the response options are balanced and include a ‘don't’ know option: strongly disagree, disagree, slightly disagree, slightly agree, agree, strongly agree, don't know. The achieved number of respondents for the survey was 2057, with 1045 in the best practice condition, 511 in the positive skew condition and 501 in the negative skew condition.

All respondents are presented with the following 10 statements.

(1) Coronavirus is a bioweapon developed by China to destroy the West.*
(2) Muslims are spreading the virus as an attack on Western values.*
(3) The reason the government brought in lockdown rules limiting people's movements was to keep people safe and stop the spread of coronavirus.
(4) Lockdown is a plot by environmental activists to control the rest of us.*
(5) The coronavirus vaccine will contain microchips to control the people.*
(6) The main reason for developing a vaccine for coronavirus is to help people stay healthy, not to control them in some way.
(7) The idea that coronavirus is spread by the 5 G mobile network is nonsense.
(8) The United Nations (UN) and World Health Organisation (WHO) have manufactured the virus to take global control.*
(9) Jews have created the virus to collapse the economy for financial gain.*
(10) Coronavirus was not deliberately created by anyone. It started in China when the disease spread from animals (such as bats) to humans.

Six of the 10 statements cover the range of themes covered in the original 30 specific coronavirus conspiracy statements used by Freeman et al. (2020a): malevolent actors creating the virus, using the virus, and controlling vaccination against the virus. Statements included in the survey claim that coronavirus was deliberately created, or purposely spread, for various malevolent reasons by Jews, China, the United Nations (UN)/World Health Organisation (WHO), or Muslims. Also included was a statement on environmental activists causing the ‘lockdown’ response to the virus and a statement accusing vaccine makers of embedding microchips in the vaccine to achieve control over the population. Four of the 10 items included in the survey are non-conspiracy statements: official or true statements relating to the cause of spread of the virus, using the virus, and vaccination against the virus. Specifically, they included statements that the virus was not deliberatively created but emerged from animals in China, that lockdown policy and vaccine creation are driven by public health concerns, and that 5 G infrastructure does not cause the virus.

We use a similar version of the wording used in the Freeman et al. (2020a) survey to introduce the question to respondents: ‘Coronavirus has been a shock and lives have changed dramatically. At times of crisis, people may think of lots of different explanations for what is occurring. We now present you with a wide range of views about Coronavirus – some have a lot of evidence supporting them, others have no evidence supporting them. Please indicate how much you agree or disagree with each view’.^4^

#### Measures of compliance

We ask respondents to indicate, on a 1–7 scale running from ‘not at all’ to ‘completely’, how much they adhere to current guidelines, and how much they will comply with future guidelines.^5^ We ask three specific current compliance questions: on social distancing, handwashing and mask wearing, asking respondents how often they have adhered in the preceding seven days (always, often, sometimes, not very often, or never). For future compliance, we asked respondents whether they intended to: download a coronavirus app, isolate if contacted and requested to do so, take a vaccine if developed, stop friends and family taking a vaccine, and wear a face mask (yes definitely, yes probably, no probably not, or no definitely not).

#### Measures of trust

We ask respondents to indicate the extent to which they trust the government, doctors, scientists, and the WHO (never, rarely, sometimes, often, or always).

### Pre-registration, ethics, and data transparency

Full details of this study were pre-registered before any data was collected, including precise wording of the hypotheses, survey details, and analytical strategy.^6^ Ethical approval for this study was granted by the Ethics Committee of Queen's University Belfast's School of History, Anthropology, Philosophy and Politics. Data analysis was conducted using SPSS (26). The dataset used in this study is publicly available, along with the code required to replicate the analysis (SPSS syntax file).^7^ The study was funded using resources from Queen's University Belfast.

## Results

### Skewed positive v. skewed negative: testing Hypothesis 1

**Fig. 1.**
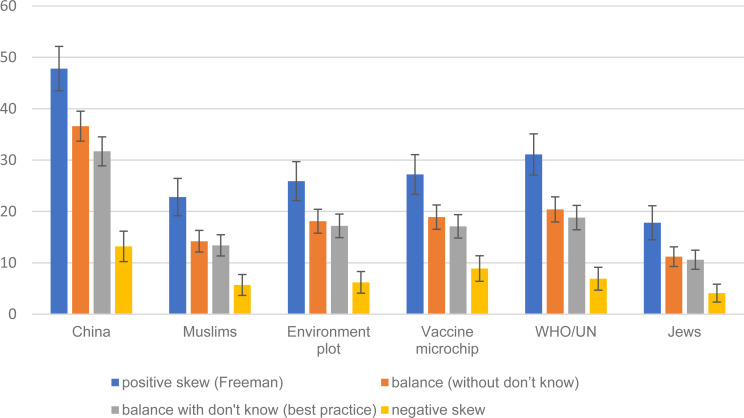
Support for Covid 19 conspiracy statements, by type of response options. Confirming Hypothesis 1, for all items in the negative skew condition the % is lower than the % in the positive skew condition at 0.001 level of statistical significance. Confirming Hypothesis 2, for all items in the best practice condition the % is lower than the % in the positive skew condition at 0.001 level of statistical significance. Confirming Hypothesis 3, for all items the balance without don't know % falls between the negative skew % and positive skew %; all differences with negative skew significant at 0.001 and all differences with positive skew significant at 0.001 of statistical significance. 95% confidence intervals are reported.

For each of the six conspiracy statements, we compare the percentage agreeing with the statement across two of the experimental conditions (positively skewed and negatively skewed response options). As reported in columns 1 and 4 in Table 1, and as visually illustrated in Fig. 1, the percentage agreeing with each statement is far smaller in the negative than the positive skew condition, and is often negligible in absolute terms. On average over the six items the percentage in the negative skew column is only one quarter of the percentage in the positive skew column. This finding demonstrates the massive effect that response option skew has on the observed support for conspiracy statements, confirming Hypothesis 1.^8^

**Table 1.** Agreement with coronavirus conspiracy statements (%)

	Positive skew (Freeman)	Balanced with DK excluded^a^	Balanced with DK (best practice)^b^	Negative skew^c^
Coronavirus is a bioweapon developed by China to destroy the West	47.8	36.6	31.7	13.2
Muslims are spreading the virus as an attack on Western values	22.8	14.2	13.4	5.7
Lockdown is a plot by environmental activists to control the rest of us	25.9	18.1	17.2	6.2
The coronavirus vaccine will contain microchips to control the people	27.2	18.9	17.1	8.9
The United Nations (UN) and World Health Organisation (WHO) have manufactured the virus to take global control	31.1	20.4	18.8	6.9
Jews have created the virus to collapse the economy for financial gain	17.8	11.2	10.6	4.1

a Confirming Hypothesis 3, for all items the % falls between the negative skew % and positive skew %; all differences with negative skew statistically significant at 0.001 level and all differences with positive skew statistically significant at 0.001.

b Confirming Hypothesis 2, for all items the % is lower than the % in the positive skew column at 0.001 level of statistical significance.

c Confirming Hypothesis 1, for all items the % is lower than the % in the positive skew column at 0.001 level of statistical significance.

### Best practice v. skewed: testing Hypothesis 2

For each of the individual coronavirus conspiracy items we compare our advocated ‘best practice’ condition (balanced response set and a ‘don't know’ option) to the positive skew condition as used by Freeman et al. (2020a). As reported in columns 1 and 3 in Table 1, and as visually illustrated in Fig. 1, in all six cases the prevalence of support is lower, hence confirming Hypothesis 2. On average the prevalence of support in the best practice condition is only five eigths – 62.3% – the size of the prevalence reported in the positive skew (Freeman et al., 2020a) condition. For example, the positive skew method would indicate that 23% agreement with the claim that ‘Muslims are spreading the virus as an attack on Western values’. On our best practice estimate, this proportion is 13%.^9^

### Best practice minus ‘don't knows’ v. skewed: testing Hypothesis 3

To focus in all conditions on those who provided a substantive response, we exclude the ‘don't know’ respondents from the ‘best practice’ condition. For each conspiracy statement we compare the percentage agreeing across all three conditions. As reported in columns 1, 2, and 4 in Table 1, and as visually illustrated in Fig. 1, for all six items, the percentage agreeing in the ‘balanced, excluding don't knows’ group is lower than the positive skew and higher than the negative skew conditions, hence confirming hypothesis 3.

### Conspiracies and compliance: testing Hypothesis 4

Using the respondents in our ‘best practice’ condition (*n* = 1045) we investigate whether a reliable scale of attitudes to coronavirus conspiracies can be generated from responses to the full set of 10 statements. Principal components factor analysis and investigation of Cronbach's alpha revealed that responses to the 10 items did not reduce to a single dimension. As often happens with balanced scales (e.g. Schriesheim & Eisenbach, 1995; Zhang, Noor, & Savalei, 2016), two dimensions emerged according to whether the items are worded in a positive or negative direction. This attests to the power of acquiescence which, by making some respondents agree with both pro- and anti-conspiracy statements, weakens the expected negative correlation between the two. This underlines the case for balanced scales if the goal is a less biased estimate of the overall level of support for conspiracy beliefs. However, it means that further work needs to be done to develop an effective unidimensional measure from a battery made up of pro- and anti-conspiracy statements.

Partly for that reason, and partly to provide a more direct comparison with the unbalanced scale results reported by Freeman et al. (2020a), we focus here on responses to the six pro-conspiracy statements and use those to generate – for all three experimental conditions – a summed scale running from anti- to pro-coronavirus conspiracies. We recalibrated these scales to run from 0 to 1.

To test hypothesis 4, we compare this coronavirus conspiracy beliefs scale to measures of adherence to guidelines on coronavirus. We first describe the distributions on the adherence measures. As reported in Table 2, few respondents indicate that they are not adhering, or planning not to adhere, to coronavirus guidelines. Most of the variation on the 7-point scales is between the mid-point and ‘completely’ adhering. As reported in Table 3, public adherence to both social distancing and handwashing is high, with most of the variation being between ‘always’ and ‘sometimes’. On wearing face masks (which had not yet become mandatory at the time of fieldwork), there are almost similar proportions at both ends of the scale: 30% say ‘always’ and 27% say ‘never’. Table 4 reports intended adherence with new guidelines. One third say they would not download a coronavirus app onto their phone. Only one in 10 say they would not self-isolate if contacted and asked to do so. One in seven say they would not take a vaccine, or would stop family and friends taking a vaccine, while only 8% say they do not intend to wear a mask when it becomes mandatory to do so.

**Table 2.** Following recommendations from government to prevent the spread of the coronavirus…?

	Not at all						Completely	
	1	2	3	4	5	6	7	dk
Are you following recommendations?	0.7	0.7	3.2	7.9	21.3	30.9	34.3	1.0
Will you follow future Recommendations…?	0.9	0.4	3.0	8.1	19.7	31.1	34.2	2.6

**Table 3.** In the last 7 days, how often…?

	Always	Often	Some-times	Not very often	Never	dk
Have you stayed at least one metre away from other people when outside your home?	50.5	28.4	13.4	3.8	2.8	1.1
Did you wash your hands with soap and water straight away after returning home from a public place?	57.8	20.3	14.0	4.5	2.8	0.6
Did you wear a face mask when you were in a shop or on public transport?	30.4	15.9	13.5	9.6	27.0	3.5

**Table 4.** Following specific new guidelines

	Yes, definitely	Yes, probably	No, probably not	No, definitely not	dk
If advised by government, would you download a contact tracing app onto your phone to help control the spread of the coronavirus?	26.9	31.4	18.7	15.6	7.4
If you were contacted by the NHS and urged to self-isolate (stay at home) because you had been in contact with someone with Coronavirus, would you do so?	61.3	26.0	7.3	2.5	2.8
If a new vaccine were to be developed that could prevent Coronavirus, would you take it?	50.9	26.8	9.1	5.6	7.7
Would you try to stop family and friends from taking a new Coronavirus vaccine?	4.9	8.8	19.5	59.1	7.7
From July 24 everyone must by law wear a face mask when they are in a shop or on public transport. Will you do so?	71.4	17.9	4.7	3.3	2.7

**Fig. 2.**
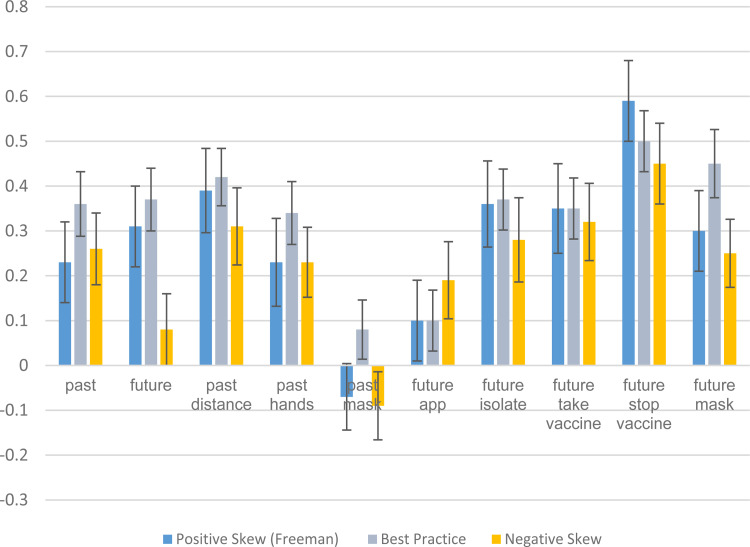
Correlations between Covid conspiracy support and adherence. Consistent with Hypothesis 4, there is a higher positive correlation between conspiracy beliefs and adherence when the best practice measure is used than when the positive skew (Freeman et al.) measure (or negative skew) measure is used (in 16 out of 18 cases, two cases are identical). However, the differences are not typically statistically significant. Hence, the Hypothesis 4 is only weakly supported.

For each of these 10 adherence items we calculate a Pearson correlation between the item and the 0–1 coronavirus conspiracy scale, broken down by experimental condition.^10^ The correlations are reported in Fig. 2. First, we compare the correlations in the best practice condition to the correlations in the positive skew (Freeman et al. (2020a)) condition. In two of the 10 comparisons the correlations are identical. In seven out of the other eight correlation comparisons there is a higher positive correlation in the best practice condition than in the positive skew condition. Next, we compare correlations in the best practice condition to correlations in the negative skew condition. The former yields a higher positive correlation than the latter in nine out of 10 cases. Hence, overall, 16 out of 18 comparable cases (89%) are consistent with hypothesis 4. However, the observed differences in correlation strength are typically not statistically significant (as shown in Fig. 2). Hence, Hypothesis 4 is weakly supported. Insofar as stronger correlations denote better measurement, our best practice response scale looks superior than the Freeman et al. (2020a) approach but the lack of significant differences is an indication that the difference is not large.

### Conspiracies, trust, and compliance: testing Hypothesis 5

To test hypothesis 5, we focus on the role of public trust in institutions and professions. In relation to the distribution of opinion on each our four trust measures, doctors are most trusted (39% always trust), followed by scientists (21%) and the WHO (14%), and the government is least trusted (7%) (Table 5). The four trust items did not produce a strong uni-dimensional scale, with a Cronbach's alpha below perhaps the most commonly cited cut-off point of 0.7 (Nunnally, 1978) and substantial scale improvement was indicated if items were deleted. Hence, we use the distinct trust items in our analysis.

**Table 5.** To what extent do you trust…?

	Never trust	Rarely trust	Some-times trust	Often trust	Always trust	dk
The government	12.4	22.5	33.2	24.1	6.5	1.3
Doctors	1.4	4.2	15.2	38.6	39.3	1.3
Scientists	2.6	7.4	22.0	44.8	21.0	2.3
World Health Organisation (WHO)	5.3	11.5	25.2	37.8	14.3	6.0

**Fig. 3.**
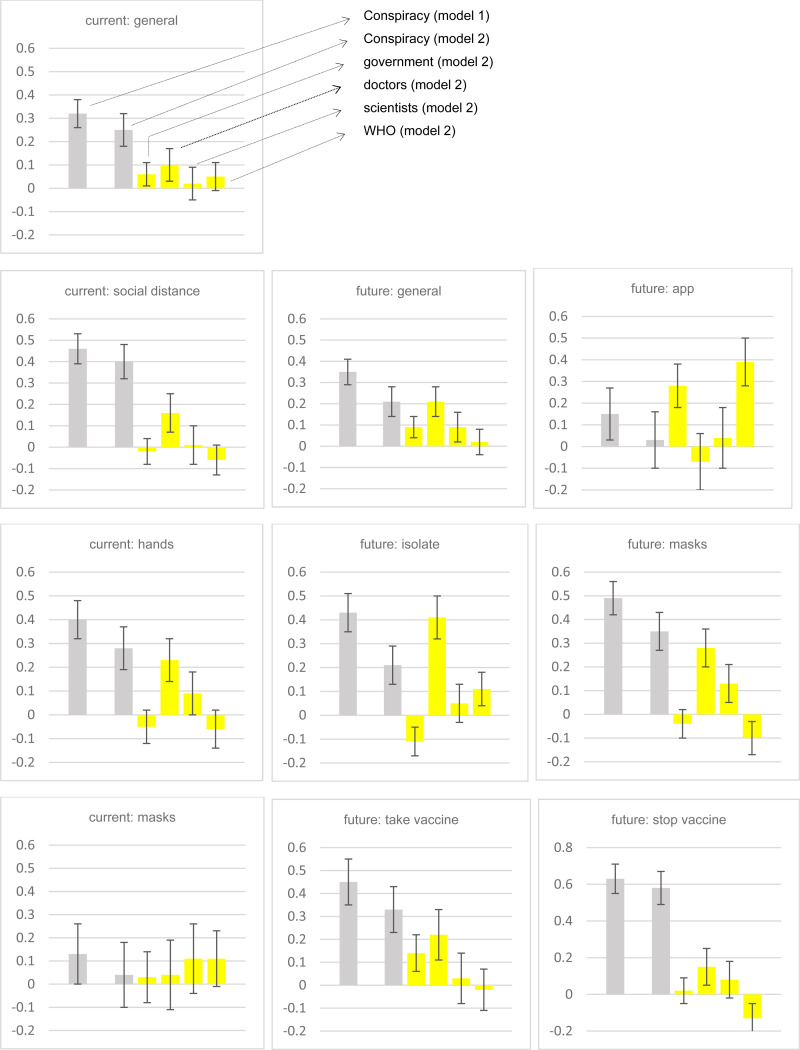
Effect of pro-conspiracy beliefs on adherence, without (model 1) and with (model 2) distrust variables. *Note*: all predictor and outcome measures are coded 0-1, co-efficients and 95% confidence intervals reported from OLS regressions (see online Supplementary materials for full details). For some adherence measures (e.g. future: isolate) trust variables reduce conspiracy effect, and not others (e.g. stop vaccine). Partial confirmation of Hypothesis 5.

For each of the 10 adherence outcome measures we run two OLS regressions: in model 1 we include only the pro-conspiracy beliefs scale and in model 2 we additionally include the four distrust variables. All outcome measures and predictors were coded 0–1 to facilitate easy interpretation of the maximum effect of each variable, and all outcome measures were coded such that higher scores indicated greater non-adherence. As reported in Fig. 3 (see online Supplementary materials for full regression results), in relation to outcome measure ‘future: general’ we see that a general intention not to adhere to future Covid 19 guidelines is strongly predicted by belief in conspiracy theories. Comparing respondents at the most pro-conspiracy end of the conspiracy beliefs scale to those with lowest belief in conspiracy theories is associated with a difference of over one third of the range of responses on the non-adherence measure. However, the conspiracy beliefs effect reduces substantially (down to one fifth of the response range), and statistically significantly, upon the introduction of the distrust measures: conspiracy beliefs and distrust in doctors are equally powerful predictors. A more dramatic effect is shown for the ‘future: isolate’ outcome measure (an initially strong conspiracy beliefs effect is reduced and outperformed by distrust in doctors) and ‘future: app’ (an initially strong conspiracy beliefs effect is reduced and outperformed by distrust in government and the WHO). Conversely, in relation to ‘current: general’, ‘current: social distance’ and most especially in relation to ‘stop vaccine’ the effect of pro-conspiracy beliefs initially was, and remained, very strong. Thus, Hypothesis 5 is only partially confirmed: only in relation to four out of the 10 adherence outcomes measures (future: general, future: isolate, future: app and current: masks) does the introduction of the trust variables either render the conspiracy predictor non-statistically significant or generate a statistically significant reduction in its effect.

### Which population groups believe conspiracies and mistrust?

**Fig. 4.**
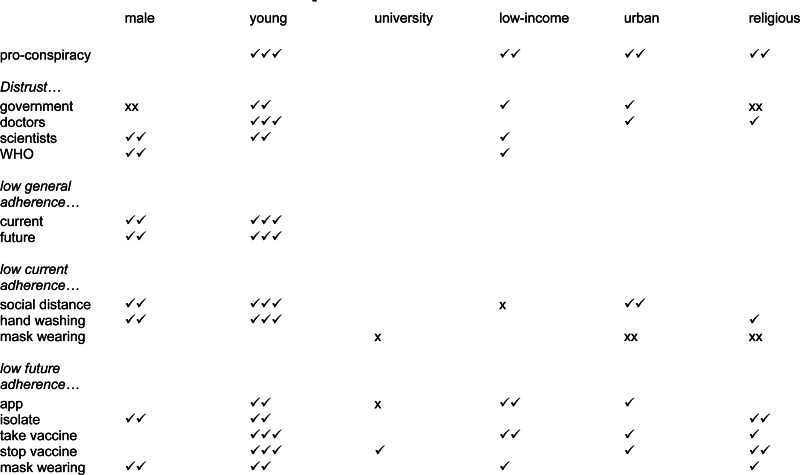
Socio-demographic bases of support for Covid conspiracies, distrust, and non-adherence. *Note 1*: derived from regression analyses reported in online Supplementary materials *Note 2*: ✓ indicates that people with the demographic trait have a deficiency on the outcome measure, i.e. young people are more likely to believe in conspiracies and are more distrusting of government, etc. The more ✓s the stronger the relationship. ✓ statistically significant at 0.05, ✓✓ statistically significant at 0.001, ✓✓✓ statistically significant at 0.001 and co-efficient at least double the size of the next largest co-efficient. These distinctions are to facilitate a quick sense of the results; please see full details in the online Supplementary materials. *Note 3*: x indicates the opposite relationship, i.e. males are *less* distrusting of government.

If, as just described, believing coronavirus conspiracies and mistrusting institutions/professions are both associated with non-adherence, it is important to identify which particular groups in the population are most prone to having these traits as this may be helpful for any consideration of how to effectively respond. To shine an exploratory light,^11^ we run OLS regressions with the measures of conspiracy beliefs, distrust, and adherence as outcomes and the following as predictors: gender (male/female), age (young: under 40, mid: 40−59, old: 60 or older), education (university versus non-university), income (low, middle, or high), urban residency *v.* not, and religiosity (self-described as religious versus not). The full results are reported in the online Supplementary materials, but for simplicity we illustrate the key findings in Fig. 4.

In short, being young is by far the most important demographic predictor of conspiracy belief, mistrust, and non-adherence. Also, being low-income, urban and religious predicts being supportive of conspiracies. Being male is a strong predictor of six of the 10 adherence outcomes, and being low-income, urban and religious predict several adherence measures.^12^ The effect of high education varies, being related to greater mask wearing and app downloading, but also to a higher likelihood of trying to stop others taking a vaccine.^13^

## Discussion

We agree with Freeman et al. (2020a) that it is important to study the extent and possible impact of coronavirus conspiracy beliefs. To ignore or underestimate those beliefs, and their possible consequences for compliance with public health measures, would be a mistake. However, to overestimate these beliefs and their impact is also a mistake. Good methodological practice is all the more important in the middle of a pandemic. Publishing and publicising those overestimates risks two serious negative consequences. It may further stigmatise certain groups who are blamed by conspiracy theorists, and it may encourage the re-direction of scarce public resources towards countering the over-estimated conspiracy views instead of countering more plausible and potent hindrances to compliant public health behaviour (McManus et al., 2020; Sutton & Douglas, 2020). Indeed, on the estimation of prevalence, ‘misleading evidence may be more damaging than no evidence at all’ (McManus et al., 2020).

In this paper, we have empirically demonstrated that the response options used by Freeman et al. (2020a) lead to significantly inflated estimates of prevalence – sometimes almost double the level recorded by the more conventional approach of a balanced response scale with a don't know option. Our findings on the inflated estimates provided by the Freeman *et al*. (2020a) approach echo those of Sutton and Douglas (2020), whose similar experiment with a convenience sample used three of the same survey items as in our study. Using our own ‘best practice’ measures, and drawing on evidence from a high-quality representative sample of the population, we have provided more accurate estimates of the prevalence of support for coronavirus conspiracies. We strongly advocate that future research avoids the problems of over-estimation associated with the Freeman et al. (2020a) approach.^14^

It is worth emphasising that our intent here is to estimate accurately the incidence of coronavirus conspiracy beliefs rather than to downplay or minimise the importance of the problem. While our estimates are substantially lower than those provided by Freeman et al. (2020a), they still indicate that non-trivial proportions of the population support coronavirus conspiracy beliefs. In that sense, our results represent a partial replication of the findings of Freeman et al. (2020a). We disagree with them about the extent of, but not the existence of, conspiratorial thinking in this vital area.

Our suggested ‘best practice’ approach is not only about providing a more accurate estimate of the distribution of conspiracy beliefs. Better measurement also means less bias in estimating the correlation of those beliefs with other substantive variables, notably current and future adherence to the guidelines and rules that are key to controlling the virus. Our hypothesis on that front was only weakly confirmed, however. As is typically the case, correlational results are more resistant to question wording effects than are the distributions of individual variables (Schuman & Presser, 1996).

Of course, estimates of any *causal* effect of conspiratorial beliefs on compliance requires not just good measurement but also a move beyond bivariate correlations. By taking a step in that direction with controls for trust in various actors, we have provided a more restrained estimate of the potential effect of conspiracy beliefs on adherence. This was just one step. The coefficients for both conspiracy beliefs and trust may still be inflated by omitted variable bias. Confident causal inference would require much more fully specified models than were permitted by our primarily methodological survey. This only reinforces the need for caution before claiming or even implying that a correlation or regression coefficient indicates the kind of causal effect that policymakers should beware of. We acknowledge, however, that the bivariate relationship between conspiracy beliefs and adherence observed by Freeman et al. (2020a) is partially replicated in our analysis even when controlling for trust (see also Allington, Duffy, Wessely, Dhavan, and Rubin, 2020 and Roozenbeek et al., 2020).

In what has unavoidably been a critical piece, we want to end on a constructive and collegial note. While we have disagreed on methodological grounds with the measurement choices made by Freeman et al. (2020a) and do not wish our findings to be misconstrued as ‘exaggerated’ (Freeman et al., 2020c), we do not contest that the phenomenon of coronavirus conspiracy beliefs is important, should be taken seriously, and is worthy of further systematic study. In this spirit, we look forward to engaging in further work on the shared objective of identifying how widespread support for conspiracies is and identifying the extent to which such support may causally determine behaviour on adherence to virus guidelines.

## Footnotes

† The notes appear after the main text.

## Notes

1 A commonplace example relates to the questionnaires used to measure students' evaluation of teaching. These often contain batteries of positively worded statements (rather than an equal balance of positively and negatively worded statements) with which the respondents are asked to agree or disagree (see e.g. Kember & Ginns, 2012).

2 Note that some authors defend the use of batteries of positively worded items when generating multi-item scales (Matlock, Turner, & Gitchel, 2018; see also Schriesheim & Eisenbach, 1995; and Zhang et al., 2016).

3 Sutton and Douglas (2020) report results of an experiment on a convenience sample which focused on asking three of the Freeman et al. (2020a) survey items with different response option sets. Their study provides provisional evidence of over-estimation arising from using the positively skewed response set used by Freeman et al. (2020a) compared to a balanced nine-point scale and a balanced five-point scale. Though motivated by similar concerns, our study is independent of the Sutton and Douglas (2020) study, and was pre-registered prior to the publication of the Sutton and Douglas (2020) correspondence.

4 This 10-item battery was the final question in the survey. Directly after answering the question, respondents were presented with the information that all six conspiracy statements have no evidence supporting them, and respondents were advised to use reliable sources of information such as the NHS and the WHO.

5 The measures of compliance and measures of trust used in our survey are similar to, but not exact replications of, compliance and trust items asked in the Freeman et al. (2020a, 2020b, 2020c) study. Note that all our compliance and trust questions include a ‘don't know’ option.

6 The study was pre-registered with the Open Science Framework (OSF), date stamped 15 July 3.06pm (on the ‘Attitudes to Covid 19’ project website). Pre-registration occurred once the very final version of the questionnaire had been agreed with the survey company. Data collection commenced on 16 July and ended on 19 July. Link to pre-registration: https://osf.io/uhjqm/

7 Available on the ‘Attitudes to Covid 19’ project website on OSF. Data and coding in STATA format are also available. Link to data: https://osf.io/uhjqm/

8 Note that the % agreement in our positive skew experimental condition is very similar to the % agreement in the positively skewed original Freeman et al. (2020a) study. On the China, Jew and Muslim items, the estimates are within three percentage points of each other. This high degree of similarity suggests that the approaches to generating a good quality representative sample are consistent across our study and the original Freeman et al. (2020a). In contrast, in the study by Sutton and Douglas (2020) that focused on these three particular items, the % agreement in their positive skew condition was extremely low (only 2 or 3% on the Muslim and Jew items), which is indicative of their sample being more akin to a convenience sample than a representative sample (and hence useful for experimentally identifying differences but not for providing reliable prevalence estimates).

9 We also compared our three experimental conditions in terms of the number of conspiracy beliefs each respondent supported. Respondents in the positive skew (Freeman et al., 2020a, 2020b, 2020c) condition support 1.73 conspiracy beliefs, those in the negative skew condition support 0.45, and those in our ‘best practice’ (balanced response options, with don't know) condition support 1.09 (all mean differences statistically significant at the 0.001 level). The problem of inflated estimates of agreement at item level accumulates over the full set of items, leading to large over-estimates of consistent pro-conspiracy beliefs when using the positive skew (Freeman et al. (2020a, 2020b, 2020c) approach): 14.6% of respondents in the positive skew condition agreed with all six pro-conspiracy statements compared to only 3.5% in our ‘best practice’ condition (and 0.6% in the negative skew condition).

10 The substantive results are the same if, given the ordinal nature of some of the adherence items, we calculate Kendall's tau-b correlations instead.

11 We do not have pre-registered specific hypotheses on the direction of the relationships.

12 See Allington et al. (2020) for similar findings on age and gender.

13 While an effective response may vary depending on the particular adherence behaviour under consideration, we speculate that one constructive approach may be to identify creative ways of demonstrating to young people the trustworthiness of their own doctor. That doctor is then in a stronger position to impart the message that conspiracies are indefensible and that following Covid guidelines is necessary for the health of self and others.

14 The Freeman et al. findings have, according to the Altmetric information on the journal website as of 26 November 2020, been reported by 140 news outlets around the world, conveying an inflated sense of the prevalence of conspiracy beliefs to a very broad audience.

## Supplementary material

For supplementary material accompanying this paper visit https://doi.org/10.1017/S0033291720005164. click here to view supplementary material
