# Supplementary material for: Coronavirus conspiracy beliefs, mistrust, and compliance: taking measurement seriously
Source: Psychol Med. 2020 Dec 10:1–11. doi: 10.1017/S0033291720005164 (PMC7844213; doi:10.1017/S0033291720005164)
Supplement: Supplementary file 1 [file S0033291720005164sup.zip › S0033291720005164sup001.docx]

**Regression for conspiracy beliefs (derived from best practice items)**

| **Model Summary** | | | | |
| --- | --- | --- | --- | --- |
| Model | R | R Square | Adjusted R Square | Std. Error of the Estimate |
| 1 | .419^a^ | .176 | .166 | .20860 |
| a. Predictors: (Constant), Religious, Mid_Income, Urban_City, male, UniOrStill, midVrest, Low_Income, youngVrest | | | | |

| **Coefficients^a^** | | | | | | |
| --- | --- | --- | --- | --- | --- | --- |
| Model | | Unstandardized Coefficients | | Standardized Coefficients | t | Sig. |
|  |  | B | Std. Error | Beta |  |  |
| 1 | (Constant) | .043 | .023 |  | 1.837 | .067 |
|  | male | .024 | .016 | .053 | 1.502 | .134 |
|  | midVrest | .080 | .021 | .164 | 3.864 | .000 |
|  | youngVrest | .164 | .021 | .349 | 7.996 | .000 |
|  | UniOrStill | -.007 | .018 | -.014 | -.386 | .700 |
|  | Mid_Income | .036 | .019 | .077 | 1.848 | .065 |
|  | Low_Income | .077 | .021 | .151 | 3.619 | .000 |
|  | Urban_City | .073 | .018 | .144 | 4.018 | .000 |
|  | Religious | .080 | .018 | .154 | 4.360 | .000 |
| a. Dependent Variable: sumCCgroup3balanced | | | | | | |

**Regression for distrust government**

| **Model Summary** | | | | |
| --- | --- | --- | --- | --- |
| Model | R | R Square | Adjusted R Square | Std. Error of the Estimate |
| 1 | .215^a^ | .046 | .042 | .27224 |
| a. Predictors: (Constant), Religious, male, Low_Income, Urban_City, midVrest, UniOrStill, Mid_Income, youngVrest | | | | |

| **Coefficients^a^** | | | | | | |
| --- | --- | --- | --- | --- | --- | --- |
| Model | | Unstandardized Coefficients | | Standardized Coefficients | t | Sig. |
|  |  | B | Std. Error | Beta |  |  |
| 1 | (Constant) | .499 | .018 |  | 28.296 | .000 |
|  | male | -.042 | .013 | -.075 | -3.334 | .001 |
|  | midVrest | .057 | .016 | .099 | 3.627 | .000 |
|  | youngVrest | .089 | .017 | .150 | 5.334 | .000 |
|  | UniOrStill | -.012 | .014 | -.019 | -.819 | .413 |
|  | Mid_Income | -.015 | .015 | -.027 | -1.007 | .314 |
|  | Low_Income | .048 | .016 | .078 | 2.950 | .003 |
|  | Urban_City | .034 | .014 | .056 | 2.393 | .017 |
|  | Religious | -.074 | .015 | -.116 | -5.083 | .000 |
| a. Dependent Variable: distrust_govt | | | | | | |

**Regression for distrust doctors**

| **Model Summary** | | | | |
| --- | --- | --- | --- | --- |
| Model | R | R Square | Adjusted R Square | Std. Error of the Estimate |
| 1 | .240^a^ | .057 | .053 | .22467 |
| a. Predictors: (Constant), Religious, male, Low_Income, Urban_City, midVrest, UniOrStill, Mid_Income, youngVrest | | | | |

| **Coefficients^a^** | | | | | | |
| --- | --- | --- | --- | --- | --- | --- |
| Model | | Unstandardized Coefficients | | Standardized Coefficients | t | Sig. |
|  |  | B | Std. Error | Beta |  |  |
| 1 | (Constant) | .159 | .015 |  | 10.943 | .000 |
|  | male | -.009 | .010 | -.020 | -.879 | .380 |
|  | midVrest | .031 | .013 | .065 | 2.389 | .017 |
|  | youngVrest | .106 | .014 | .215 | 7.684 | .000 |
|  | UniOrStill | .007 | .012 | .013 | .565 | .572 |
|  | Mid_Income | -.014 | .012 | -.029 | -1.101 | .271 |
|  | Low_Income | .011 | .013 | .023 | .851 | .395 |
|  | Urban_City | .044 | .012 | .088 | 3.795 | .000 |
|  | Religious | .026 | .012 | .049 | 2.135 | .033 |
| a. Dependent Variable: distrust_doctors | | | | | | |

**Regression for distrust scientists**

| **Model Summary** | | | | |
| --- | --- | --- | --- | --- |
| Model | R | R Square | Adjusted R Square | Std. Error of the Estimate |
| 1 | .170^a^ | .029 | .025 | .23891 |
| a. Predictors: (Constant), Religious, male, Mid_Income, Urban_City, UniOrStill, midVrest, Low_Income, youngVrest | | | | |

| **Coefficients^a^** | | | | | | |
| --- | --- | --- | --- | --- | --- | --- |
| Model | | Unstandardized Coefficients | | Standardized Coefficients | t | Sig. |
|  |  | B | Std. Error | Beta |  |  |
| 1 | (Constant) | .258 | .016 |  | 16.219 | .000 |
|  | male | .041 | .011 | .085 | 3.687 | .000 |
|  | midVrest | -.011 | .014 | -.022 | -.778 | .437 |
|  | youngVrest | .053 | .015 | .103 | 3.582 | .000 |
|  | UniOrStill | -.019 | .012 | -.036 | -1.495 | .135 |
|  | Mid_Income | .013 | .013 | .026 | .958 | .338 |
|  | Low_Income | .037 | .014 | .070 | 2.564 | .010 |
|  | Urban_City | .020 | .013 | .037 | 1.550 | .121 |
|  | Religious | .013 | .013 | .024 | 1.014 | .311 |
| a. Dependent Variable: distrust_scientists | | | | | | |

**Regression for distrust WHO**

| **Model Summary** | | | | |
| --- | --- | --- | --- | --- |
| Model | R | R Square | Adjusted R Square | Std. Error of the Estimate |
| 1 | .150^a^ | .022 | .018 | .26218 |
| a. Predictors: (Constant), Religious, male, Low_Income, Urban_City, midVrest, UniOrStill, Mid_Income, youngVrest | | | | |

| **Coefficients^a^** | | | | | | |
| --- | --- | --- | --- | --- | --- | --- |
| Model | | Unstandardized Coefficients | | Standardized Coefficients | t | Sig. |
|  |  | B | Std. Error | Beta |  |  |
| 1 | (Constant) | .340 | .018 |  | 19.277 | .000 |
|  | male | .065 | .012 | .123 | 5.244 | .000 |
|  | midVrest | -.005 | .015 | -.009 | -.311 | .756 |
|  | youngVrest | .010 | .016 | .018 | .613 | .540 |
|  | UniOrStill | -.005 | .014 | -.009 | -.384 | .701 |
|  | Mid_Income | -.012 | .015 | -.022 | -.809 | .419 |
|  | Low_Income | .039 | .016 | .066 | 2.406 | .016 |
|  | Urban_City | -.006 | .014 | -.010 | -.422 | .673 |
|  | Religious | .007 | .014 | .011 | .470 | .639 |
| a. Dependent Variable: distrust_WHO | | | | | | |

**Regression for current: general**

| **Model Summary** | | | | |
| --- | --- | --- | --- | --- |
| Model | R | R Square | Adjusted R Square | Std. Error of the Estimate |
| 1 | .336^a^ | .113 | .109 | .18726 |
| a. Predictors: (Constant), Religious, male, Mid_Income, Urban_City, UniOrStill, midVrest, Low_Income, youngVrest | | | | |

| **Coefficients^a^** | | | | | | |
| --- | --- | --- | --- | --- | --- | --- |
| Model | | Unstandardized Coefficients | | Standardized Coefficients | t | Sig. |
|  |  | B | Std. Error | Beta |  |  |
| 1 | (Constant) | .111 | .012 |  | 9.135 | .000 |
|  | male | .048 | .009 | .121 | 5.555 | .000 |
|  | midVrest | .040 | .011 | .097 | 3.712 | .000 |
|  | youngVrest | .145 | .011 | .344 | 12.682 | .000 |
|  | UniOrStill | -.004 | .010 | -.010 | -.424 | .671 |
|  | Mid_Income | .006 | .010 | .014 | .542 | .588 |
|  | Low_Income | -.008 | .011 | -.018 | -.700 | .484 |
|  | Urban_City | .013 | .010 | .031 | 1.388 | .165 |
|  | Religious | -.002 | .010 | -.004 | -.197 | .843 |
| a. Dependent Variable: GenCurrCompNeg01 | | | | | | |

**Regression for future: general**

| **Model Summary** | | | | |
| --- | --- | --- | --- | --- |
| Model | R | R Square | Adjusted R Square | Std. Error of the Estimate |
| 1 | .344^a^ | .118 | .114 | .18751 |
| a. Predictors: (Constant), Religious, male, Low_Income, Urban_City, midVrest, UniOrStill, Mid_Income, youngVrest | | | | |

| **Coefficients^a^** | | | | | | |
| --- | --- | --- | --- | --- | --- | --- |
| Model | | Unstandardized Coefficients | | Standardized Coefficients | t | Sig. |
|  |  | B | Std. Error | Beta |  |  |
| 1 | (Constant) | .110 | .012 |  | 9.001 | .000 |
|  | male | .069 | .009 | .173 | 7.895 | .000 |
|  | midVrest | .052 | .011 | .127 | 4.806 | .000 |
|  | youngVrest | .144 | .012 | .340 | 12.462 | .000 |
|  | UniOrStill | -.005 | .010 | -.011 | -.490 | .624 |
|  | Mid_Income | -.020 | .010 | -.048 | -1.899 | .058 |
|  | Low_Income | -.003 | .011 | -.007 | -.255 | .799 |
|  | Urban_City | -.002 | .010 | -.004 | -.177 | .860 |
|  | Religious | -.018 | .010 | -.039 | -1.765 | .078 |
| a. Dependent Variable: GenFutCompNeg01 | | | | | | |

**Regression for current: distance**

| **Model Summary** | | | | |
| --- | --- | --- | --- | --- |
| Model | R | R Square | Adjusted R Square | Std. Error of the Estimate |
| 1 | .354^a^ | .125 | .122 | .23208 |
| a. Predictors: (Constant), Religious, male, Low_Income, Urban_City, midVrest, UniOrStill, Mid_Income, youngVrest | | | | |

| **Coefficients^a^** | | | | | | |
| --- | --- | --- | --- | --- | --- | --- |
| Model | | Unstandardized Coefficients | | Standardized Coefficients | t | Sig. |
|  |  | B | Std. Error | Beta |  |  |
| 1 | (Constant) | .095 | .015 |  | 6.292 | .000 |
|  | male | .035 | .011 | .070 | 3.230 | .001 |
|  | midVrest | .082 | .013 | .161 | 6.146 | .000 |
|  | youngVrest | .194 | .014 | .367 | 13.533 | .000 |
|  | UniOrStill | .000 | .012 | .000 | -.017 | .987 |
|  | Mid_Income | -.040 | .013 | -.078 | -3.100 | .002 |
|  | Low_Income | -.035 | .014 | -.063 | -2.492 | .013 |
|  | Urban_City | .040 | .012 | .074 | 3.320 | .001 |
|  | Religious | .020 | .012 | .036 | 1.640 | .101 |
| a. Dependent Variable: CurrDistNeg01 | | | | | | |

**Regression for current: hands**

| **Model Summary** | | | | |
| --- | --- | --- | --- | --- |
| Model | R | R Square | Adjusted R Square | Std. Error of the Estimate |
| 1 | .340^a^ | .115 | .112 | .24685 |
| a. Predictors: (Constant), Religious, male, Mid_Income, Urban_City, UniOrStill, midVrest, Low_Income, youngVrest | | | | |

| **Coefficients^a^** | | | | | | |
| --- | --- | --- | --- | --- | --- | --- |
| Model | | Unstandardized Coefficients | | Standardized Coefficients | t | Sig. |
|  |  | B | Std. Error | Beta |  |  |
| 1 | (Constant) | .059 | .016 |  | 3.713 | .000 |
|  | male | .081 | .011 | .154 | 7.104 | .000 |
|  | midVrest | .069 | .014 | .128 | 4.886 | .000 |
|  | youngVrest | .189 | .015 | .340 | 12.534 | .000 |
|  | UniOrStill | -.019 | .013 | -.035 | -1.524 | .128 |
|  | Mid_Income | -.017 | .014 | -.032 | -1.263 | .207 |
|  | Low_Income | .003 | .015 | .005 | .182 | .855 |
|  | Urban_City | .015 | .013 | .026 | 1.175 | .240 |
|  | Religious | .030 | .013 | .050 | 2.280 | .023 |
| a. Dependent Variable: CurrHandsNeg01 | | | | | | |

**Regression for current: masks**

| **Model Summary** | | | | |
| --- | --- | --- | --- | --- |
| Model | R | R Square | Adjusted R Square | Std. Error of the Estimate |
| 1 | .167^a^ | .028 | .024 | .40056 |
| a. Predictors: (Constant), Religious, male, Mid_Income, Urban_City, UniOrStill, midVrest, Low_Income, youngVrest | | | | |

| **Coefficients^a^** | | | | | | |
| --- | --- | --- | --- | --- | --- | --- |
| Model | | Unstandardized Coefficients | | Standardized Coefficients | t | Sig. |
|  |  | B | Std. Error | Beta |  |  |
| 1 | (Constant) | .524 | .026 |  | 19.844 | .000 |
|  | male | .031 | .019 | .038 | 1.666 | .096 |
|  | midVrest | .010 | .023 | .012 | .417 | .677 |
|  | youngVrest | .012 | .025 | .014 | .500 | .617 |
|  | UniOrStill | -.061 | .021 | -.071 | -2.935 | .003 |
|  | Mid_Income | -.011 | .022 | -.014 | -.508 | .611 |
|  | Low_Income | -.003 | .024 | -.003 | -.125 | .900 |
|  | Urban_City | -.079 | .021 | -.088 | -3.747 | .000 |
|  | Religious | -.090 | .022 | -.098 | -4.181 | .000 |
| a. Dependent Variable: CurrMaskNeg01 | | | | | | |

**Regression for future: app**

| **Model Summary** | | | | |
| --- | --- | --- | --- | --- |
| Model | R | R Square | Adjusted R Square | Std. Error of the Estimate |
| 1 | .190^a^ | .036 | .032 | .34314 |
| a. Predictors: (Constant), Religious, male, Mid_Income, Urban_City, UniOrStill, midVrest, Low_Income, youngVrest | | | | |

| **Coefficients^a^** | | | | | | |
| --- | --- | --- | --- | --- | --- | --- |
| Model | | Unstandardized Coefficients | | Standardized Coefficients | t | Sig. |
|  |  | B | Std. Error | Beta |  |  |
| 1 | (Constant) | .303 | .023 |  | 13.119 | .000 |
|  | male | .015 | .016 | .021 | .901 | .368 |
|  | midVrest | .094 | .020 | .131 | 4.574 | .000 |
|  | youngVrest | .121 | .022 | .164 | 5.551 | .000 |
|  | UniOrStill | -.039 | .018 | -.053 | -2.156 | .031 |
|  | Mid_Income | .007 | .019 | .009 | .346 | .729 |
|  | Low_Income | .071 | .021 | .091 | 3.323 | .001 |
|  | Urban_City | .056 | .018 | .074 | 3.059 | .002 |
|  | Religious | -.027 | .019 | -.034 | -1.411 | .158 |
| a. Dependent Variable: FutAppNeg01 | | | | | | |

**Regression for future: isolate**

| **Model Summary** | | | | |
| --- | --- | --- | --- | --- |
| Model | R | R Square | Adjusted R Square | Std. Error of the Estimate |
| 1 | .253^a^ | .064 | .060 | .24114 |
| a. Predictors: (Constant), Religious, male, Low_Income, Urban_City, midVrest, UniOrStill, Mid_Income, youngVrest | | | | |

| **Coefficients^a^** | | | | | | |
| --- | --- | --- | --- | --- | --- | --- |
| Model | | Unstandardized Coefficients | | Standardized Coefficients | t | Sig. |
|  |  | B | Std. Error | Beta |  |  |
| 1 | (Constant) | .076 | .016 |  | 4.839 | .000 |
|  | male | .046 | .011 | .093 | 4.116 | .000 |
|  | midVrest | .063 | .014 | .122 | 4.483 | .000 |
|  | youngVrest | .134 | .015 | .253 | 8.997 | .000 |
|  | UniOrStill | .000 | .013 | -.001 | -.024 | .981 |
|  | Mid_Income | -.016 | .013 | -.031 | -1.166 | .244 |
|  | Low_Income | -.014 | .015 | -.026 | -.965 | .335 |
|  | Urban_City | -.003 | .013 | -.006 | -.246 | .806 |
|  | Religious | .044 | .013 | .077 | 3.378 | .001 |
| a. Dependent Variable: FutIsolNeg01 | | | | | | |

**Regression for future: take vaccine**

| **Model Summary** | | | | |
| --- | --- | --- | --- | --- |
| Model | R | R Square | Adjusted R Square | Std. Error of the Estimate |
| 1 | .306^a^ | .094 | .089 | .28236 |
| a. Predictors: (Constant), Religious, male, Mid_Income, Urban_City, UniOrStill, midVrest, Low_Income, youngVrest | | | | |

| **Coefficients^a^** | | | | | | |
| --- | --- | --- | --- | --- | --- | --- |
| Model | | Unstandardized Coefficients | | Standardized Coefficients | t | Sig. |
|  |  | B | Std. Error | Beta |  |  |
| 1 | (Constant) | .073 | .019 |  | 3.870 | .000 |
|  | male | -.025 | .014 | -.041 | -1.819 | .069 |
|  | midVrest | .114 | .017 | .186 | 6.822 | .000 |
|  | youngVrest | .191 | .018 | .304 | 10.763 | .000 |
|  | UniOrStill | .021 | .015 | .033 | 1.399 | .162 |
|  | Mid_Income | .016 | .016 | .026 | .977 | .329 |
|  | Low_Income | .088 | .017 | .135 | 5.039 | .000 |
|  | Urban_City | .035 | .015 | .054 | 2.323 | .020 |
|  | Religious | .042 | .016 | .062 | 2.697 | .007 |
| a. Dependent Variable: FutTakeVNeg01 | | | | | | |

**Regression for future: stop vaccine**

| **Model Summary** | | | | |
| --- | --- | --- | --- | --- |
| Model | R | R Square | Adjusted R Square | Std. Error of the Estimate |
| 1 | .364^a^ | .133 | .129 | .27261 |
| a. Predictors: (Constant), Religious, male, Low_Income, Urban_City, midVrest, UniOrStill, Mid_Income, youngVrest | | | | |

| **Coefficients^a^** | | | | | | |
| --- | --- | --- | --- | --- | --- | --- |
| Model | | Unstandardized Coefficients | | Standardized Coefficients | t | Sig. |
|  |  | B | Std. Error | Beta |  |  |
| 1 | (Constant) | .003 | .018 |  | .178 | .859 |
|  | male | .023 | .013 | .040 | 1.798 | .072 |
|  | midVrest | .104 | .016 | .172 | 6.449 | .000 |
|  | youngVrest | .217 | .017 | .349 | 12.622 | .000 |
|  | UniOrStill | .038 | .014 | .062 | 2.665 | .008 |
|  | Mid_Income | .038 | .016 | .063 | 2.447 | .015 |
|  | Low_Income | .027 | .017 | .042 | 1.604 | .109 |
|  | Urban_City | .046 | .015 | .072 | 3.154 | .002 |
|  | Religious | .065 | .015 | .098 | 4.327 | .000 |
| a. Dependent Variable: FutStopVNeg01 | | | | | | |

REGRESSION

/MISSING LISTWISE

/STATISTICS COEFF OUTS R ANOVA

/CRITERIA=PIN(.05) POUT(.10)

/NOORIGIN

/DEPENDENT FutMaskNeg01

/METHOD=ENTER male midVrest youngVrest UniOrStill Mid_Income Low_Income Urban_City Religious.

**Regression for future: masks**

| **Model Summary** | | | | |
| --- | --- | --- | --- | --- |
| Model | R | R Square | Adjusted R Square | Std. Error of the Estimate |
| 1 | .234^a^ | .055 | .051 | .24188 |
| a. Predictors: (Constant), Religious, male, Mid_Income, Urban_City, UniOrStill, midVrest, Low_Income, youngVrest | | | | |

| **Coefficients^a^** | | | | | | |
| --- | --- | --- | --- | --- | --- | --- |
| Model | | Unstandardized Coefficients | | Standardized Coefficients | t | Sig. |
|  |  | B | Std. Error | Beta |  |  |
| 1 | (Constant) | .026 | .016 |  | 1.642 | .101 |
|  | male | .061 | .011 | .123 | 5.445 | .000 |
|  | midVrest | .036 | .014 | .070 | 2.544 | .011 |
|  | youngVrest | .110 | .015 | .208 | 7.334 | .000 |
|  | UniOrStill | .005 | .013 | .010 | .418 | .676 |
|  | Mid_Income | .032 | .014 | .062 | 2.358 | .018 |
|  | Low_Income | .029 | .015 | .052 | 1.965 | .050 |
|  | Urban_City | -.012 | .013 | -.022 | -.948 | .343 |
|  | Religious | .027 | .013 | .048 | 2.091 | .037 |
| a. Dependent Variable: FutMaskNeg01 | | | | | | |
