# Supplementary material for: Coronavirus conspiracy beliefs, mistrust, and compliance: taking measurement seriously
Source: Psychol Med. 2020 Dec 10:1–11. doi: 10.1017/S0033291720005164 (PMC7844213; doi:10.1017/S0033291720005164)
Supplement: Supplementary file 1 [file S0033291720005164sup.zip › S0033291720005164sup002.docx]

**Regression results for Hypothesis 4, relating to Figure 3.**

**Regression for current: general**

| **Model Summary** | | | | |
| --- | --- | --- | --- | --- |
| Model | R | R Square | Adjusted R Square | Std. Error of the Estimate |
| 1 | .358^a^ | .128 | .127 | .18844 |
| 2 | .407^b^ | .166 | .160 | .18480 |
| a. Predictors: (Constant), sumCCgroup3balanced | | | | |
| b. Predictors: (Constant), sumCCgroup3balanced, distrust_govt, distrust_WHO, distrust_doctors, distrust_scientists | | | | |

| **Coefficients^a^** | | | | | | |
| --- | --- | --- | --- | --- | --- | --- |
| Model | | Unstandardized Coefficients | | Standardized Coefficients | t | Sig. |
|  |  | B | Std. Error | Beta |  |  |
| 1 | (Constant) | .136 | .010 |  | 14.147 | .000 |
|  | sumCCgroup3balanced | .320 | .031 | .358 | 10.299 | .000 |
| 2 | (Constant) | .069 | .017 |  | 4.106 | .000 |
|  | sumCCgroup3balanced | .248 | .034 | .278 | 7.258 | .000 |
|  | distrust_govt | .064 | .026 | .088 | 2.439 | .015 |
|  | distrust_doctors | .095 | .036 | .113 | 2.620 | .009 |
|  | distrust_scientists | .024 | .037 | .029 | .651 | .515 |
|  | distrust_WHO | .050 | .030 | .066 | 1.668 | .096 |
| a. Dependent Variable: GenCurrCompNeg01 | | | | | | |

**Regression for future: general**

| **Model Summary** | | | | |
| --- | --- | --- | --- | --- |
| Model | R | R Square | Adjusted R Square | Std. Error of the Estimate |
| 1 | .375^a^ | .140 | .139 | .19250 |
| 2 | .504^b^ | .254 | .249 | .17982 |
| a. Predictors: (Constant), sumCCgroup3balanced | | | | |
| b. Predictors: (Constant), sumCCgroup3balanced, distrust_govt, distrust_WHO, distrust_doctors, distrust_scientists | | | | |

| **Coefficients^a^** | | | | | | |
| --- | --- | --- | --- | --- | --- | --- |
| Model | | Unstandardized Coefficients | | Standardized Coefficients | t | Sig. |
|  |  | B | Std. Error | Beta |  |  |
| 1 | (Constant) | .127 | .010 |  | 12.804 | .000 |
|  | sumCCgroup3balanced | .346 | .032 | .375 | 10.797 | .000 |
| 2 | (Constant) | .027 | .016 |  | 1.662 | .097 |
|  | sumCCgroup3balanced | .210 | .033 | .228 | 6.291 | .000 |
|  | distrust_govt | .085 | .026 | .112 | 3.291 | .001 |
|  | distrust_doctors | .213 | .035 | .242 | 6.009 | .000 |
|  | distrust_scientists | .090 | .036 | .105 | 2.511 | .012 |
|  | distrust_WHO | .022 | .029 | .027 | .735 | .462 |
| a. Dependent Variable: GenFutCompNeg01 | | | | | | |

**Regression for current: distance**

| **Model Summary** | | | | |
| --- | --- | --- | --- | --- |
| Model | R | R Square | Adjusted R Square | Std. Error of the Estimate |
| 1 | .424^a^ | .180 | .179 | .22242 |
| 2 | .448^b^ | .201 | .195 | .22020 |
| a. Predictors: (Constant), sumCCgroup3balanced | | | | |
| b. Predictors: (Constant), sumCCgroup3balanced, distrust_govt, distrust_WHO, distrust_doctors, distrust_scientists | | | | |

| **Coefficients^a^** | | | | | | |
| --- | --- | --- | --- | --- | --- | --- |
| Model | | Unstandardized Coefficients | | Standardized Coefficients | t | Sig. |
|  |  | B | Std. Error | Beta |  |  |
| 1 | (Constant) | .104 | .011 |  | 9.181 | .000 |
|  | sumCCgroup3balanced | .462 | .037 | .424 | 12.575 | .000 |
| 2 | (Constant) | .108 | .020 |  | 5.375 | .000 |
|  | sumCCgroup3balanced | .403 | .041 | .370 | 9.868 | .000 |
|  | distrust_govt | -.020 | .031 | -.022 | -.625 | .532 |
|  | distrust_doctors | .162 | .043 | .158 | 3.742 | .000 |
|  | distrust_scientists | .014 | .044 | .014 | .319 | .750 |
|  | distrust_WHO | -.057 | .036 | -.061 | -1.576 | .115 |
| a. Dependent Variable: CurrDistNeg01 | | | | | | |

**Regression for current: hands**

| **Model Summary** | | | | |
| --- | --- | --- | --- | --- |
| Model | R | R Square | Adjusted R Square | Std. Error of the Estimate |
| 1 | .345^a^ | .119 | .118 | .24631 |
| 2 | .413^b^ | .170 | .165 | .23967 |
| a. Predictors: (Constant), sumCCgroup3balanced | | | | |
| b. Predictors: (Constant), sumCCgroup3balanced, distrust_govt, distrust_WHO, distrust_doctors, distrust_scientists | | | | |

| **Coefficients^a^** | | | | | | |
| --- | --- | --- | --- | --- | --- | --- |
| Model | | Unstandardized Coefficients | | Standardized Coefficients | t | Sig. |
|  |  | B | Std. Error | Beta |  |  |
| 1 | (Constant) | .096 | .013 |  | 7.615 | .000 |
|  | sumCCgroup3balanced | .401 | .041 | .345 | 9.867 | .000 |
| 2 | (Constant) | .089 | .022 |  | 4.094 | .000 |
|  | sumCCgroup3balanced | .284 | .044 | .244 | 6.398 | .000 |
|  | distrust_govt | -.053 | .034 | -.056 | -1.553 | .121 |
|  | distrust_doctors | .233 | .047 | .213 | 4.965 | .000 |
|  | distrust_scientists | .093 | .048 | .087 | 1.947 | .052 |
|  | distrust_WHO | -.060 | .039 | -.060 | -1.522 | .128 |
| a. Dependent Variable: CurrHandsNeg01 | | | | | | |

**Regression for current: masks**

| **Model Summary** | | | | |
| --- | --- | --- | --- | --- |
| Model | R | R Square | Adjusted R Square | Std. Error of the Estimate |
| 1 | .076^a^ | .006 | .004 | .38424 |
| 2 | .158^b^ | .025 | .018 | .38158 |
| a. Predictors: (Constant), sumCCgroup3balanced | | | | |
| b. Predictors: (Constant), sumCCgroup3balanced, distrust_govt, distrust_WHO, distrust_doctors, distrust_scientists | | | | |

| **Coefficients^a^** | | | | | | |
| --- | --- | --- | --- | --- | --- | --- |
| Model | | Unstandardized Coefficients | | Standardized Coefficients | t | Sig. |
|  |  | B | Std. Error | Beta |  |  |
| 1 | (Constant) | .387 | .020 |  | 19.481 | .000 |
|  | sumCCgroup3balanced | .130 | .064 | .076 | 2.043 | .041 |
| 2 | (Constant) | .302 | .035 |  | 8.609 | .000 |
|  | sumCCgroup3balanced | .035 | .071 | .020 | .490 | .624 |
|  | distrust_govt | .034 | .055 | .024 | .623 | .534 |
|  | distrust_doctors | .038 | .076 | .024 | .507 | .612 |
|  | distrust_scientists | .108 | .077 | .069 | 1.405 | .160 |
|  | distrust_WHO | .114 | .063 | .079 | 1.819 | .069 |
| a. Dependent Variable: CurrMaskNeg01 | | | | | | |

**Regression for future: app**

| **Model Summary** | | | | |
| --- | --- | --- | --- | --- |
| Model | R | R Square | Adjusted R Square | Std. Error of the Estimate |
| 1 | .091^a^ | .008 | .007 | .35773 |
| 2 | .407^b^ | .166 | .160 | .32900 |
| a. Predictors: (Constant), sumCCgroup3balanced | | | | |
| b. Predictors: (Constant), sumCCgroup3balanced, distrust_govt, distrust_WHO, distrust_doctors, distrust_scientists | | | | |

| **Coefficients^a^** | | | | | | |
| --- | --- | --- | --- | --- | --- | --- |
| Model | | Unstandardized Coefficients | | Standardized Coefficients | t | Sig. |
|  |  | B | Std. Error | Beta |  |  |
| 1 | (Constant) | .375 | .019 |  | 19.911 | .000 |
|  | sumCCgroup3balanced | .146 | .061 | .091 | 2.380 | .018 |
| 2 | (Constant) | .109 | .030 |  | 3.591 | .000 |
|  | sumCCgroup3balanced | .030 | .063 | .019 | .471 | .638 |
|  | distrust_govt | .278 | .048 | .216 | 5.828 | .000 |
|  | distrust_doctors | -.071 | .066 | -.048 | -1.071 | .285 |
|  | distrust_scientists | .039 | .068 | .027 | .576 | .565 |
|  | distrust_WHO | .386 | .055 | .287 | 7.057 | .000 |
| a. Dependent Variable: FutAppNeg01 | | | | | | |

**Regression for future: isolate**

| **Model Summary** | | | | |
| --- | --- | --- | --- | --- |
| Model | R | R Square | Adjusted R Square | Std. Error of the Estimate |
| 1 | .377^a^ | .142 | .141 | .24014 |
| 2 | .550^b^ | .302 | .298 | .21714 |
| a. Predictors: (Constant), sumCCgroup3balanced | | | | |
| b. Predictors: (Constant), sumCCgroup3balanced, distrust_govt, distrust_WHO, distrust_doctors, distrust_scientists | | | | |

| **Coefficients^a^** | | | | | | |
| --- | --- | --- | --- | --- | --- | --- |
| Model | | Unstandardized Coefficients | | Standardized Coefficients | t | Sig. |
|  |  | B | Std. Error | Beta |  |  |
| 1 | (Constant) | .078 | .012 |  | 6.383 | .000 |
|  | sumCCgroup3balanced | .433 | .040 | .377 | 10.910 | .000 |
| 2 | (Constant) | .029 | .020 |  | 1.465 | .143 |
|  | sumCCgroup3balanced | .206 | .040 | .179 | 5.111 | .000 |
|  | distrust_govt | -.105 | .031 | -.112 | -3.401 | .001 |
|  | distrust_doctors | .413 | .043 | .381 | 9.695 | .000 |
|  | distrust_scientists | .049 | .043 | .046 | 1.126 | .261 |
|  | distrust_WHO | .110 | .035 | .113 | 3.109 | .002 |
| a. Dependent Variable: FutIsolNeg01 | | | | | | |

**Regression for future: take vaccine**

| **Model Summary** | | | | |
| --- | --- | --- | --- | --- |
| Model | R | R Square | Adjusted R Square | Std. Error of the Estimate |
| 1 | .346^a^ | .119 | .118 | .27768 |
| 2 | .418^b^ | .175 | .169 | .26962 |
| a. Predictors: (Constant), sumCCgroup3balanced | | | | |
| b. Predictors: (Constant), sumCCgroup3balanced, distrust_govt, distrust_WHO, distrust_doctors, distrust_scientists | | | | |

| **Coefficients^a^** | | | | | | |
| --- | --- | --- | --- | --- | --- | --- |
| Model | | Unstandardized Coefficients | | Standardized Coefficients | t | Sig. |
|  |  | B | Std. Error | Beta |  |  |
| 1 | (Constant) | .138 | .015 |  | 9.546 | .000 |
|  | sumCCgroup3balanced | .446 | .046 | .346 | 9.624 | .000 |
| 2 | (Constant) | .039 | .025 |  | 1.565 | .118 |
|  | sumCCgroup3balanced | .331 | .050 | .257 | 6.559 | .000 |
|  | distrust_govt | .136 | .040 | .127 | 3.441 | .001 |
|  | distrust_doctors | .223 | .054 | .184 | 4.140 | .000 |
|  | distrust_scientists | .027 | .055 | .023 | .496 | .620 |
|  | distrust_WHO | -.017 | .045 | -.015 | -.367 | .714 |
| a. Dependent Variable: FutTakeVNeg01 | | | | | | |

**Regression for future: masks**

| **Model Summary** | | | | |
| --- | --- | --- | --- | --- |
| Model | R | R Square | Adjusted R Square | Std. Error of the Estimate |
| 1 | .456^a^ | .208 | .207 | .21580 |
| 2 | .548^b^ | .300 | .295 | .20351 |
| a. Predictors: (Constant), sumCCgroup3balanced | | | | |
| b. Predictors: (Constant), sumCCgroup3balanced, distrust_govt, distrust_WHO, distrust_doctors, distrust_scientists | | | | |

| **Coefficients^a^** | | | | | | |
| --- | --- | --- | --- | --- | --- | --- |
| Model | | Unstandardized Coefficients | | Standardized Coefficients | t | Sig. |
|  |  | B | Std. Error | Beta |  |  |
| 1 | (Constant) | .024 | .011 |  | 2.165 | .031 |
|  | sumCCgroup3balanced | .493 | .036 | .456 | 13.766 | .000 |
| 2 | (Constant) | .009 | .018 |  | .480 | .632 |
|  | sumCCgroup3balanced | .353 | .038 | .327 | 9.315 | .000 |
|  | distrust_govt | -.041 | .029 | -.046 | -1.396 | .163 |
|  | distrust_doctors | .278 | .040 | .275 | 6.952 | .000 |
|  | distrust_scientists | .130 | .041 | .131 | 3.180 | .002 |
|  | distrust_WHO | -.097 | .033 | -.107 | -2.934 | .003 |
| a. Dependent Variable: FutMaskNeg01 | | | | | | |

**Regression for future: stop vaccine**

| **Model Summary** | | | | |
| --- | --- | --- | --- | --- |
| Model | R | R Square | Adjusted R Square | Std. Error of the Estimate |
| 1 | .505^a^ | .255 | .254 | .24595 |
| 2 | .529^b^ | .280 | .275 | .24244 |
| a. Predictors: (Constant), sumCCgroup3balanced | | | | |
| b. Predictors: (Constant), sumCCgroup3balanced, distrust_govt, distrust_WHO, distrust_doctors, distrust_scientists | | | | |

| **Coefficients^a^** | | | | | | |
| --- | --- | --- | --- | --- | --- | --- |
| Model | | Unstandardized Coefficients | | Standardized Coefficients | t | Sig. |
|  |  | B | Std. Error | Beta |  |  |
| 1 | (Constant) | .059 | .013 |  | 4.599 | .000 |
|  | sumCCgroup3balanced | .632 | .041 | .505 | 15.336 | .000 |
| 2 | (Constant) | .051 | .023 |  | 2.251 | .025 |
|  | sumCCgroup3balanced | .575 | .046 | .460 | 12.628 | .000 |
|  | distrust_govt | .023 | .035 | .023 | .659 | .510 |
|  | distrust_doctors | .153 | .048 | .128 | 3.171 | .002 |
|  | distrust_scientists | .075 | .049 | .063 | 1.509 | .132 |
|  | distrust_WHO | -.129 | .041 | -.119 | -3.170 | .002 |
| a. Dependent Variable: FutStopVNeg01 | | | | | | |
